# First results of a sepsis protocol at Diadema State Hospital

**DOI:** 10.1186/cc10160

**Published:** 2011-06-22

**Authors:** FM Gazoni, ILS Braga, E Giordanni, LS Vendrame

**Affiliations:** 1 Hospital Estadual de Diadema, Diadema - SP, Brazil

## Introduction

Sepsis is an important cause of death at Diadema State Hospital, therefore a sepsis protocol was designed.

## Objective

To reduce sepsis prevalence, morbidity, the mortality rate and its high cost.

## Methods

An audit was conducted in the period of April to September 2010 with data collected through hospital records.

## Results

Sixty-three patients were enrolled. Analyzed was each item of the package of 6 hours according to the designed protocol, including total adherence to the package of 6 hours, mortality of eligible patients and mortality of patients who adhered to the package of 6 hours. Of 63 patients, 28 patients were discharged and 35 evolved to death, only one case not correlated with death from septic shock. Mortality due to sepsis at our service was 56%, which is consistent with the mortality rate in Brazil (57.3%, according to ILAS) and in public hospitals (63.9%). Adherence to the package of 6 hours recommended by the SSC was only 21 of the 63 cases. Of these 21 cases, 11 patients survived and 10 died. Thirty cases of all had some compliance with the protocol of 6 hours, and of these 17 were discharged and 13 died. Disrupting the total mortality (35 cases, 56%), it was found that mortality among patients who adhered to the package of 6 hours was lower (48%) when compared with those who did not join (60%).

## Conclusion

The results show a lower mortality rate in cases where there was total adherence to the package of interventions in the first 6 hours, but we still have low level of adherence to this package (33%). The average length of stay decreased dramatically from 2008 to 2010 (73% vs. 62%) when we compared the patients who died with those who survived, which is still high but has fallen over time, surpassing the survival rates measured in other public hospitals in Brazil (data from ILAS). After these first results, improvements were made to be implemented in 2011 such as review and redrafting of the protocol flow; training different categories of professionals (technicians, nurses, physiotherapists, doctors, pharmacists); realignment with ILAS, including manager selection protocol with capacity-building and training for use of the international database for comparative analysis; review the recommendation of antimicrobials for the second focus of infection with sepsis; and regular monitoring of results, including average length of stay and mortality. The challenge now is to decrease deaths, aiming to achieve levels comparable with the best institutions in the world. In partnership with ILAS, the project SPDM against Sepsis, our team has strived to achieve this goal.

